# Evidence for Multiple Origins of De Novo Formed Vascular Smooth Muscle Cells in Pulmonary Hypertension: Challenging the Dominant Model of Pre-Existing Smooth Muscle Expansion

**DOI:** 10.3390/ijerph18168584

**Published:** 2021-08-14

**Authors:** Xuran Chu, Negah Ahmadvand, Jin-San Zhang, Werner Seeger, Saverio Bellusci, Elie El Agha

**Affiliations:** 1 Department of Internal Medicine, Universities of Giessen and Marburg Lung Center (UGMLC), Cardio-Pulmonary Institute (CPI), Member of the German Center for Lung Research (DZL), 35392 Giessen, Germany; chuxuran@gmail.com (X.C.); negah.ahmadvand@innere.med.uni-giessen.de (N.A.); werner.seeger@innere.med.uni-giessen.de (W.S.); saverio.bellusci@innere.med.uni-giessen.de (S.B.); 2 School of Pharmacy, Wenzhou Medical University, Wenzhou 325015, China; zhang_jinsan@wmu.edu.cn; 3 Department of Internal Medicine, Institute for Lung Health (ILH), Justus-Liebig University, 35392 Giessen, Germany

**Keywords:** vascular remodeling, pulmonary hypertension, vascular smooth muscle cells

## Abstract

Vascular remodeling is a prominent feature of pulmonary hypertension. This process involves increased muscularization of already muscularized vessels as well as neo-muscularization of non-muscularized vessels. The cell-of-origin of the newly formed vascular smooth muscle cells has been a subject of intense debate in recent years. Identifying these cells may have important clinical implications since it opens the door for attempts to therapeutically target the progenitor cells and/or reverse the differentiation of their progeny. In this context, the dominant model is that these cells derive from pre-existing smooth muscle cells that are activated in response to injury. In this mini review, we present the evidence that is in favor of this model and, at the same time, highlight other studies indicating that there are alternative cellular sources of vascular smooth muscle cells in pulmonary vascular remodeling.

## 1. Introduction

Pulmonary hypertension (PH) is a progressive disease defined by a resting mean pulmonary arterial pressure (mPAP) greater than or equal to 25 mmHg as assessed by right heart catheterization. One of the hallmark features of PH is the remodeling process occurring in the pulmonary vasculature, including but not limited to excessive accumulation of vascular smooth muscle cells (VSMCs), deposition of extracellular matrix (ECM) proteins, and infiltration of inflammatory cells. These pathological events lead to stiffening of the vascular walls and increased vascular resistance. When left untreated, PH can lead to right heart failure [1,2,3].

In this regard, ECM deposition is believed to occur during the early phases of PH development, indicating that it might contribute to the initiation of the disease [4]. On the other hand, the inflammatory landscape has gained increasing interest in recent years, as there seems to be a clear correlation between PH and immune system dysregulation [5,6]. Here, we will focus on the cellular origin of newly formed VSMCs in PH without disregarding the significance of other aspects of this disease such as the ones mentioned above.

The vasculature of the adult lung can be classified into three categories based on the degree of muscularization: proximal fully muscularized vessels, middle partially muscularized vessels, and distal non-muscularized vessels. During PH development, there is increased muscularization of the already muscularized proximal and middle vessels and neo-muscularization of the distal vessels. The remodeling process leads to restriction of the microcirculation and contributes profoundly to increased pulmonary vascular resistance and pressure [1,2,3]. Against this background, the cellular origin of newly formed VSMCs has gained increasing interest in recent years as it (1) contributes to the overall understanding of PH pathogenesis and (2) may help develop novel therapies targeted against culprit cells/signaling pathways in affected patients. To this end, the dominant model is that newly formed VSMCs derive from pre-existing VSMCs that expand distally along the vascular tree.

Smooth muscle cells express unique contractile proteins, ion channels, and signaling molecules compared with other types of muscle cells such as skeletal or cardiac muscle. Among the SMC markers are the proteins comprising the contractile apparatus such as alpha-smooth muscle actin (αSMA or ACTA2), smooth muscle myosin heavy chain (SMMHC; myosin heavy chain 11 or MYH11), Calponin, SM22α, and Smoothelin. However, it is important to mention that some of these SMC markers are expressed, at least transiently or at relatively lower levels, in other cell types during development, tissue repair, and/or disease states. For example, we have recently shown in the context of airway epithelial regeneration that a population of mesenchymal cells transiently acquires ACTA2 expression following naphthalene injury and contributes to club-cell replenishment in the mouse lung [7]. Moreover, epithelial cells have been shown to undergo transient epithelial-to-mesenchymal transition (EMT) during this regeneration process [8]. A third example comes from embryonic development whereby the fibroblast growth factor 10-positive (FGF10+) lineage contributes to airway SMC and VSMC formation only during a tight time window during early lung development [9,10]. Therefore, some of these markers, particularly ACTA2 that is used as the bona fide marker of SMCs, can be transiently induced in both epithelial and mesenchymal cells under various conditions. In this mini review, we provide an overview of the literature that favors the model of pre-existing VSMC expansion and, at the same time, compile evidence in support of an alternative scenario whereby other sources also contribute to de novo forming VSMCs in PH. In the next sections, the contribution of various cell types to PH-associated VSMCs will be presented and discussed.

## 2. Resident Vascular Smooth Muscle Cells

Experimental studies using hypoxia-induced PH in mice suggest that newly formed VSMCs derive from pre-existing VSMCs that initially occupy the proximal regions of the vascular tree, or at least from pre-existing ACTA2+ precursors. For instance, when several lineage tracing mouse lines were used to map the fate of pre-existing (labeled before injury) CDH5+ (cadherin 5; vascular endothelial cadherin or VE-cadherin: endothelial marker), CSPG4+ (chondroitin sulfate proteoglycan 4; neuron-glial antigen 2 or NG2: pericyte marker), PDGFRα+ (platelet-derived growth factor receptor alpha: fibroblast marker), ACTA2+ and MYH11+ cells following hypoxia or house dust mite (HDM) exposure, the major contribution to newly formed VSMCs appeared to be from ACTA2+ and MYH11+ cells [11]. The same study demonstrated that there is a global increase in the proliferation of the resident mesenchymal cell types mentioned above. The analysis focused on the vessels whose diameter was less than or equal to 35 μm, as these vessels lack any VSMCs in the absence of PH-associated remodeling. It is important to mention that labeling of endothelial cells in that study was incomplete and that the CSPG4+ lineage labeled around 20% of VSMCs under hypoxic conditions [11]. Another note is that hypoxia represents a mild form of PH in mice and does not recapitulate the severity of human pulmonary arterial hypertension (PAH), including the formation of neointima and plexiform lesions.

Another elegant work showed that the progenitors of newly formed VSMCs are discrete “primed” VSMCs occupying the (muscularized) proximal—(non-muscularized) distal borders [12]. In response to hypoxia, such primed cells sequentially undergo dedifferentiation (loss of SMMHC expression), distal migration, clonal expansion, and differentiation (reacquisition of SMMHC expression). These progenitors were suggested to be platelet-derived growth factor receptor beta-positive (PDGFRβ+) [12]. Endothelial cell and macrophage-derived platelet-derived growth factor B (*Pdgfb*) is upregulated under hypoxic conditions and seems to be important for the activation of PDGFRβ+ progenitors [13,14]. Moreover, the pluripotency factor Kruppel-like factor 4 (*Klf4*) was found to be upregulated in these cells, and it seems that its expression is important for neo-muscularization of distal pulmonary arterioles [13]. These findings are based on state-of-the-art experimental approaches and analytical tools such as genetic lineage tracing, clonal analysis, and the use of confocal imaging and 3D reconstruction of thick lung sections to analyze the “same” vessel segment in control and experimental samples.

More recently, chronic HDM treatment was combined with hypoxia exposure in mice to generate an inflammatory model that mimics PAH severity in terms of perivascular inflammation, elevated right ventricular pressures, medial thickening, and neointima formation [15]. In this model, no clonal expansion of pre-existing VSMCs was observed but rather the proliferation of random VSMCs during medial thickening. Intriguingly, the authors showed that VSMCs expressing *Notch3*, but not endothelial cells, are the cellular source of neointima in this model [15].

To sum up, these studies suggest that newly formed VSMCs in PH predominantly derive from pre-existing VSMCs located proximally. In the next sections, we present data suggesting that there are alternative cellular sources for these cells apart from proximal VSMCs.

## 3. Endothelial-to-Mesenchymal Transition (EndMT)

Endothelial cells form a monolayer of cells residing in the tunica intima (innermost layer) of blood vessels. They act as a barrier between circulating blood and various organs and tissues, thus controlling the traffic of nutrients, immune cells, cytokines, growth factors, and other substances between the two entities. Endothelial-to-mesenchymal transition (EndMT) is a process by which endothelial cells lose their endothelial phenotype and acquire mesenchymal marker expression such as ACTA2. Evidence for this phenomenon stems from early experiments involving primary cultures of endothelial cells [16,17]. Although this process has been sometimes perceived as a rare event, there is a strong notion that EndMT is an important mechanism for VSMC accumulation during PH pathogenesis. Analysis of the expression of endothelial (such as CD31, CD34, VE-cadherin, and von Willebrand factor) and mesenchymal markers (such as ACTA2) is usually used to identify cells that are transitioning between the two states.

Analysis of immunostained lung sections revealed that the endothelial markers CD31, CD34, and VE-cadherin show diffuse staining in the intima and plexiform lesions of PAH samples and sometimes co-localize with ACTA2 expression, as opposed to strong staining of the thin endothelial layer adjacent to ACTA2+ VSMCs in the non-PAH group [18]. Using a novel rat model of spontaneous PH development linked to the human *BMPR2* mutations, the expression levels of the EndMT markers Twist1 and Phospho-vimentin were found to be increased in the lungs of these rats [18]. It was also proposed that decreased *BMPR2* expression in endothelial cells induces the upregulation of high mobility group AT-hook 1 (*HMGA1*), leading to EndMT in PAH [19]. This mechanism was demonstrated using in vitro cultures of pulmonary artery endothelial cells, and the expression data were validated by immunostaining of PAH lung explants. EndMT was identified by the loss of CD31 protein expression and gain of ACTA2, SM22α, Calponin, Phospho-vimentin, and Slug expression. Interestingly, EndMT could be reversed via co-inhibition of *HMGA1* or *Slug* expression [19].

In another study, endothelial cells were labeled in vivo using *Cdh5-Cre* or *Tie2-Cre* mouse driver lines combined with the *Rosa26^mT^*^/mG^ reporter line in the context of left lung pneumonectomy followed by monocrotaline (MCT) treatment [20]. The authors reported that in this severe model of PH in mice, labeled cells contributed to neointima formation and that these cells expressed ACTA2 and SMMHC. Additionally, cells expressing both endothelial and smooth muscle markers were shown to be present in neointimal lesions of human PAH lung tissues [20].

EndMT was also reported in the sugen/hypoxia model of PH in mice. The *Cdh5-Cre* mouse driver line was used in combination with the *Rosa26^mT^*^/mG^ reporter line to isolate GFP+ CDH5- cells (EndMT cells) [21]. EndMT cells were enriched with stem cell antigen 1 (*Sca-1*) expression and displayed high proliferative and migratory properties. In vitro assays showed that EndMT cells contribute not only to VSMC-like cells but also to intimal and medial proliferation via paracrine-acting factors [21]. Finally, endothelial-like cells isolated from chronic thromboembolic pulmonary hypertension (CTEPH) patients were proposed to transit to mesenchymal phenotypes and display endothelial dysfunction when exposed to myofibroblast-like cells or their conditioned medium [22].

## 4. Argument for the Contribution of Perivascular Mesenchymal (Progenitor) Cells

During embryonic lung development in mice, arterial walls are constructed radially through a mechanism that is coordinated by two processes: One involves the successive incorporation of cells from the surrounding PDGFRβ+ mesenchyme to form the various layers, and the other one involves the invasion of the outer layer by inner-layer cells [23]. The latter process occurs via developmentally regulated cell reorientation and radial migration [23]. Such morphogenic process leads to the colonization of the tunica media of blood vessels by resident VSMCs.

**Figure 1:**
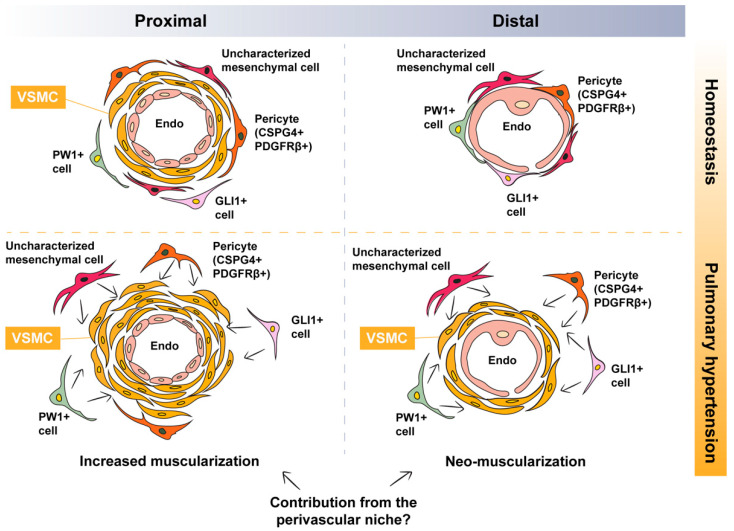
Potential contribution of perivascular mesenchymal cells to pulmonary hypertension-associated vascular smooth muscle cells. Under homeostatic conditions, proximal vessels are muscularized while distal vessels are non-muscularized. During vascular remodeling, there is increased muscularization of already muscularized vessels and neo-muscularization of non-muscularized vessels. Possible perivascular sources of VSMCs include pericytes, PW1+ cells, GLI1+ cells, and/or other uncharacterized mesenchymal cells: VSMC, vascular smooth muscle cell.

As mentioned in the previous sections, distal vessels contain a single layer of endothelial cells and are, therefore, non-muscularized under homeostatic conditions. Thus, it is plausible that the muscularization of these non-muscularized vessels involves partial, or even full, recapitulation of the developmental process described above. In the microcirculation, endothelial cells are covered by mesenchymal cells such as pericytes characterized by the expression of PDGFRβ (Figure 1). Therefore, it is likely that the remodeling process involves the activation of “local” PDGFRβ+ cells, leading to their differentiation into bona fide VSMCs. In this section, we provide an overview of the literature that is in favor of this scenario.

### 4.1. Are Pericytes a Source of PH-Associated VSMCs?

Pericytes are mesenchymal cells residing in the perivascular space where they influence various aspects of vascular biology such as vessel stability, vascular tone, and ECM deposition, to name a few (Figure 1). Pericytes are also regarded as mesenchymal stem cell (MSC)-like cells due to their trilineage differentiation potential toward adipocytes, chondrocytes, and osteoblasts [24,25,26]. It has been demonstrated that pericyte coverage is significantly increased in pulmonary arteries of human PAH lungs [27]. Moreover, primary human lung pericytes isolated based on the expression of 3G5-ganglioside antigen (*3G5*) show increased proliferative and migratory responses when exposed to conditioned medium from PAH endothelial cells compared with controls, a mechanism that involves fibroblast growth factor 2 (FGF2) and interleukin 6 (IL-6) signaling pathways [27]. Moreover, treatment of these cells with TGFβ1 leads to their transdifferentiation into smooth muscle-like cells. Increased pericyte coverage was also demonstrated in the MCT model in rats as well as the hypoxia model in mice. Finally, the *Cspg4-DsRed* reporter line showed that CSPG4+ cells indeed gain ACTA2 expression following exposure to hypoxia [27].

In a follow-up work, 3G5+ pericytes were isolated from human patients and shown to significantly express *NG2* and *PDGFRb* in addition to classical MSC markers but little or no smooth muscle markers *SM22* and SMMHC [28]. Human-derived idiopathic pulmonary arterial hypertension (IPAH) pericytes exhibit higher wound-closure, migratory, and proliferative capabilities compared with their donor counterparts. While culturing donor and IPAH pericytes in complete medium did not reveal major differences in terms of expression of contractile genes, serum starvation revealed a higher expression of *SM22*, Calponin, and a trend for ACTA2 in IPAH pericytes [28]. Moreover, lineage tracing of NG2+ cells showed an increase in the number of lineage-labeled cells and acquisition of ACTA2 expression. Blocking CXCL-12 signaling hindered pericyte accumulation in response to hypoxia in mice, and CXCR-7 mediated the excessive pericyte proliferation and migration in PAH. IPAH pericytes also displayed an exaggerated response to TGFβ1 stimulation likely due to higher expression of TGFβ receptor II [28].

### 4.2. Are Mesenchymal Stem Cells Also Involved in Vascular Remodeling?

The arterial adventitia has been described as a sonic hedgehog (SHH)-responsive domain that supports resident SCA-1+ vascular progenitor cells [29]. Although these and other adventitial cells do not express SMC marker proteins in vivo, they express transcription factors that are involved in SMC differentiation, and they readily differentiate into SMC-like cells in vitro. The perivascular region has been identified as an MSC domain in multiple human organs [30].

Glioma-associated oncogene homolog 1 (GLI1) expression has been reported to identify perivascular MSC-like cells in multiple organs [25,31] (Figure 1). These cells are capable of trilineage differentiation and are, thus, termed MSC-like cells. GLI1+ cells are believed to constitute a subset of PDGFRβ+ pericytes, and they give rise to myofibroblasts in multiple models of fibrosis, particularly in the heart, lungs, kidneys, liver, and bone marrow [31,32]. Genetic ablation of these cells attenuates fibrosis in the heart and kidneys, and their pharmacological targeting attenuates bone marrow fibrosis [31,32]. Interestingly, GLI1+ cells have been shown to contribute to VSMC-like cells in the model of wire injury to the femoral artery [33]. Adventitial GLI1+ cells displayed increased proliferation, migrated into the medial and neointimal layers, and co-expressed ACTA2 and Calponin [33]. Last but not least, the same study showed that these cells are also progenitors for osteoblast-like cells during medial and intimal calcification in chronic kidney disease in mice [33].

Lineage tracing studies have shown that during lung development, GLI1+ cells represent a population of mesenchymal progenitors [34,35]. These cells give rise to airway SMCs and VSMCs, in addition to less characterized populations of alveolar fibroblasts. It is therefore plausible that this population of MSC-like cells undergoes reprogramming during remodeling of the pulmonary vasculature, giving rise to VSMCs (Figure 1).

Another population of perivascular MSC-like cells is the PW1+ population (Figure 1). PW1 is a zinc finger protein involved in the regulation of cell cycle and cell stress responses, β-catenin stabilization, and metabolic homeostasis in addition to its requirement for the myogenic and migratory capabilities of mesoangioblasts [36]. PW1+ cells were initially identified in murine skeletal muscle, where PW1 is expressed in satellite cells and a subset of interstitial cells with myogenic potential [37]. PW1+ interstitial cells were shown to give rise to smooth and skeletal muscle in addition to adipocytes in vitro [37]. Perivascular PW1+ cells in the mouse lung were shown to express progenitor and pericyte markers; they undergo proliferation in response to hypoxia, and they contribute to VSMC formation during vascular remodeling [36] (Figure 1). It remains to be established whether the above (and other) populations of perivascular mesenchymal cells overlap and whether they converge on a common primed VSMC cell that is recruited to the tunica media and undergoes myogenic differentiation during vascular remodeling.

## 5. Insights from Other Non-Hypoxia-Driven PH Models

While chronic hypoxia is widely used to induce pulmonary vascular remodeling in mice, there are other non-hypoxia-driven alternatives. In this section, we shed light on two commonly used models and highlight their main pathophysiological features.

### 5.1. The Monocrotaline Model

Monocrotaline is a toxin extracted from the plant *Crotalaria spectabilis*. It causes injury to the pulmonary vasculature resulting in PH in rats, and it is one of the most common PH models in experimental animals. Following MCT injection, it is activated into pyrrole metabolite dehydromonocrotaline (MCTP) in the liver by cytochrome P450 monooxygenases. MCTP is a bifunctional alkylating agent that damages the vascular endothelium [38,39]. It is suggested that such damage induces pulmonary vasculitis and right ventricular hypertrophy. Furthermore, enlarged Golgi apparatus, dislocation of endothelial nitric oxide synthase (eNOS), and disturbed intracellular membrane trafficking have been observed in pulmonary endothelial cells exposed to MCT [40,41]. The loss of membrane proteins leads to activating proliferative and antiapoptotic factors and ultimately causes change to the pulmonary vasculature in MCT-treated animals [38,42]. Like hypoxia-induced PH, MCT causes increased muscularization of the pulmonary vasculature. Due to extreme endothelial damage and related interstitial inflammation, this injury model recapitulates aspects of human PAH such as neointima formation and plexiform lesions. Note that such changes are not detected in the hypoxia model in mice. In MCT-treated rats, intra-acinar vessels are first affected by the remodeling process, followed by hilar vessels due to enhanced PAP [43,44,45,46]. Due to the limited availability of transgenic rats compared with mice and since MCT induces a PAH-like phenotype in rats but not mice, the use of this model to lineage trace VSMCs has been very limited.

### 5.2. The Sugen/Hypoxia Model

Vascular endothelial growth factor (VEGF) and its receptor 2 (VEGFR2) play an essential role in pulmonary endothelial cell maintenance and survival [47,48]. SU5416 (3-[(2,4-dimethylpyrrol-5-yl) methylidenyl]-indolin-2-one) is a synthetic VEGFR2 inhibitor [49] that causes muscularization of the pulmonary vessels leading to increased vascular resistance and PH in hypoxia-exposed rats. Massive endothelial cell death and obliteration of the artery lumen by proliferated endothelial cells are observed in SU5416-treated lungs. SU5416 also leads to the development of pulmonary vascular remodeling and mild PH in the normoxia group; however, the effect on the hypoxic group is more severe and results in irreversible PH [50]. Combining suprasystemic levels of PAP with progressive plexiform-like lesions made this model suitable for studying human PAH [51,52]. This model can be applied to mice, but the severity of PH is less than in rats.

The rate of VSMC amplification is enhanced in SU5416-treated animals, revealing the association of endothelial cell VEGFR signaling and VSMC growth. However, the proliferation of VSMCs in both large and small vessels occurs transiently at the early phase of the disease and significantly diminishes at later stages. This suggests that alternative sources of VSMCs, other than pre-existing VSMCs, may contribute to the progression of pathogenicity [43,50,52,53].

## 6. Conclusions

Although the dominant view is that pre-existing VSMCs are the major structural contributors to newly formed VSMCs in hypoxia-induced PH, there is increasing evidence that other cell types are also involved in this process. Perivascular cells, in particular, seem to be a strong candidate due to their (1) anatomical location around non-muscularized vessels and (2) intrinsic progenitor-like state where they express classical markers of mesenchymal progenitors such as PDGFRβ and GLI1. Such cells do not express significant levels of VSMC proteins such as ACTA2. However, it is possible that they are still labeled using smooth muscle lineage tracing tools such as *Acta2-Cre-ERT2* since even low expression levels of Cre-ERT2 can lead to genetic recombination and labeling of the parent cells as well as their progeny. Some aspects that remain unexplored include how the different cellular sources synergistically act to generate new VSMCs, their mode of communication, and whether there is a master regulator that coordinates their relative structural contribution. Future studies involving single-cell genomics and transcriptomics, improved lineage tracing tools, and superior imaging approaches will help develop this exciting research avenue.

## Data Availability

Not applicable.
